# Cellular mechanisms and effects of IL-4 receptor blockade in experimental conjunctivitis evoked by skin inflammation

**DOI:** 10.1172/jci.insight.163495

**Published:** 2023-02-08

**Authors:** Hongwei Han, Sheila Cummings, Kai-Ting C. Shade, Jennifer Johnson, George Qian, Joseph Gans, Srinivas Shankara, Javier Escobedo, Erik Zarazinski, Renee Bodinizzo, Dinesh Bangari, Paul Bryce, Alexandra Hicks

**Affiliations:** 1 Sanofi, Immunology and Inflammation Research Therapeutic Area, Cambridge, Massachusetts, USA.; 2 Sanofi, Global Discovery Pathology, Translational In-vivo Models Platform, Cambridge, Massachusetts, USA.; 3 Sanofi, Translational Science Single Cell & Functional Genomics, Cambridge, Massachusetts, USA.; 4 Sanofi, In-vivo Research Center, Translational In-vivo Models Platform, Cambridge, Massachusetts, USA.

**Keywords:** Dermatology, Immunology, Basophils, Cytokines, Mouse models

## Abstract

Ocular surface diseases, including conjunctivitis, are recognized as common comorbidities in atopic dermatitis (AD) and occur at an increased frequency in patients with AD treated with biologics targeting IL-4 receptor α (IL-4Rα) or IL-13. However, the inflammatory mechanisms underlying this pathology are unknown. Here, we developed a potentially novel mouse model of skin inflammation–evoked conjunctivitis and showed that it is dependent on CD4^+^ T cells and basophils. Blockade of IL-4Rα partially attenuated conjunctivitis development, downregulated basophil activation, and led to a reduction in expression of genes related to type 2 cytokine responses. Together, these data suggest that an IL-4Rα/basophil axis plays a role in the development of murine allergic conjunctivitis. Interestingly, we found a significant augmentation of a number of genes that encode tear proteins and enzymes in anti–IL-4Rα–treated mice, and it may underlie the partial efficacy in this model and may represent candidate mediators of the increased frequency of conjunctivitis following dupilumab in patients with AD.

## Introduction

Atopic dermatitis (AD) is a common allergic skin disorder that often precedes the development of allergies in distal tissues (1–3). Based on the nationwide Danish registry data, the risk of selected ocular comorbidities (conjunctivitis, keratitis, and keratoconus) in adult patients with AD is significantly higher than that of the general population, suggesting a role of AD as an entry point for subsequent ocular diseases (3, 4). The findings in patients with AD also provide a framework for experimental models to evaluate the immunological mechanisms that facilitate the progression from AD and skin barrier dysfunction to ocular disorders.

Clinical trials with dupilumab, a monoclonal Ab (mAb) that inhibits IL-4 and IL-13 signaling, have demonstrated efficacy in moderate-to-severe AD (5, 6). These trials also showed an increased incidence of conjunctivitis with dupilumab treatment (6, 7), which may be indicative of a specific AD disease interaction since conjunctivitis occurs rarely in patients with asthma, chronic rhinosinusitis with nasal polyps, or eosinophilic esophagitis who are on dupilumab treatment (8). Conjunctivitis is also observed with IL-13 monotherapy (lebrikizumab or tralokinumab), suggesting that IL-13 inhibition is implicated in the pathophysiology of conjunctivitis (9–11). At present, however, predictive biomarkers, preventative treatments, defined treatment protocols, and the underlying pathophysiological mechanisms to explain the potential increased incidence of ocular surface diseases in dupilumab-treated patients with AD remain unknown. Several hypotheses have been postulated but are not being evaluated in this article, including increased IL-17 levels and *Demodex* mite colonization (12), a qualitative tear production failure (13), increased activity of the OX40 ligand in the eye (14), IL-13–related scarcity of conjunctival goblet cells accompanied by an inflammatory T cell and eosinophilic infiltrate (15), lower local dupilumab drug concentration in the eye (13), and increased eosinophils and basophils during dupilumab treatment (16, 17). There is a timely need to better understand conjunctivitis in the context of AD using both preclinical models and clinical studies.

We aimed to develop a mouse model to further understand AD-induced conjunctivitis and to dissect the possible mechanisms of conjunctivitis in patients with AD following dupilumab treatment by exploring the impact of IL-4Rα blockade. Our studies revealed that mice exposed to the model antigen OVA in the presence of the vitamin D analog MC903 to induce AD-like inflammation in the skin developed severe conjunctivitis when later challenged with the same antigen via eye drops. Using this model, we further investigated the underlying immunological mechanisms of conjunctivitis and potential candidate genes associated with conjunctivitis in patients with AD treated with dupilumab.

## Results

### A potentially novel mouse model of skin inflammation–induced conjunctivitis.

**Figure 1:**
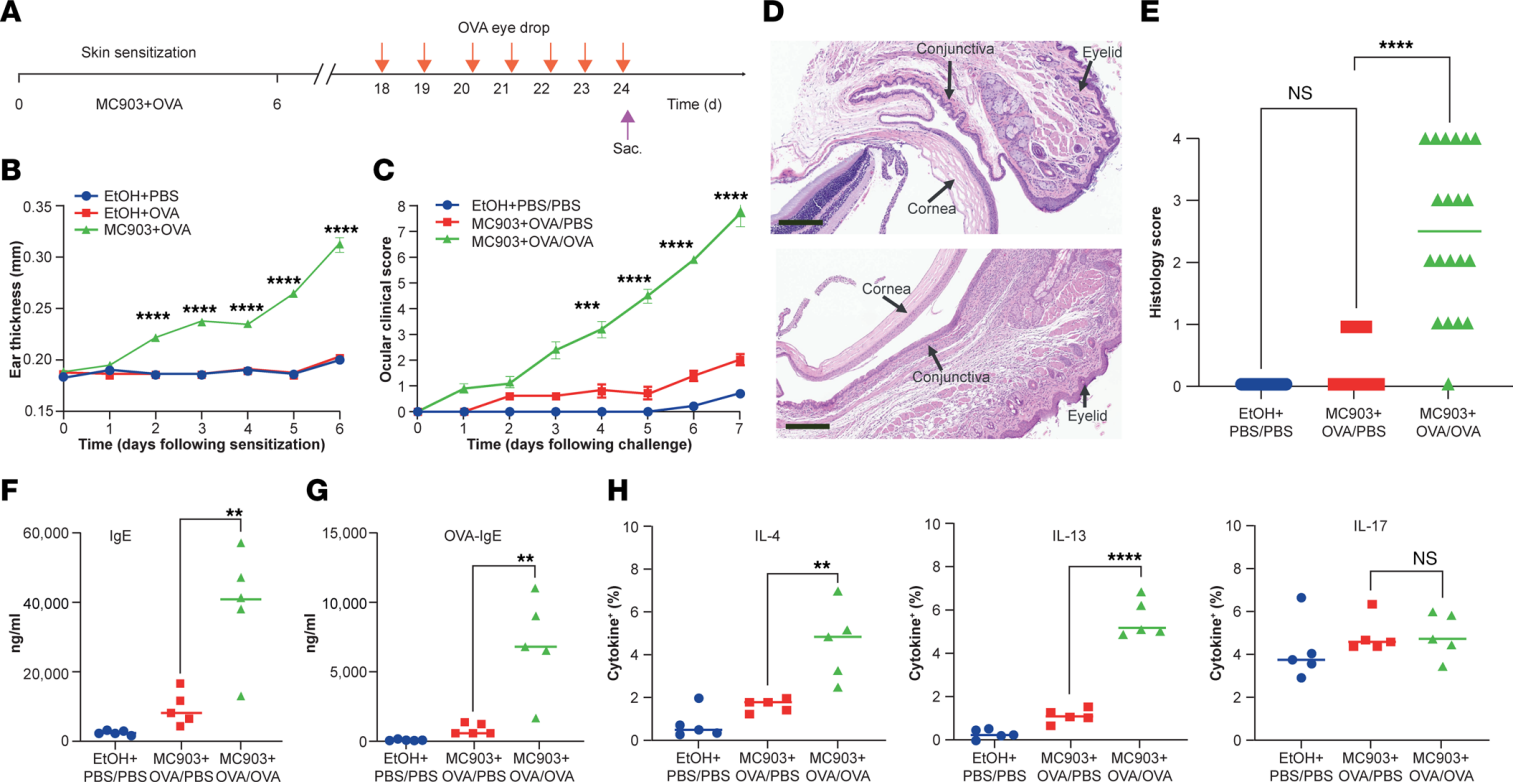
Experimental mouse model of skin inflammation–induced conjunctivitis. (**A**) Schematic of mouse conjunctivitis model. Arrows in red are time periods of administering OVA eye drop, and purple arrow indicates sacrifice. (**B**) Ear thickness of vehicle EtOH control + PBS- or OVA-treated and MC903 + OVA-treated WT mice from day 0 to day 6. Data are from 2 pooled experiments (*n* = 10). (**C**) Representative clinical signs. Data are from 2 pooled experiments (*n* = 10). Results are shown as mean ± SEM, and a 2-way ANOVA with Tukey’s post hoc test was used to determine significance for **B** and **C**. (**D**) H&E histopathology images. Upper, MC903 + OVA/PBS; lower, MC903 + OVA/OVA. Scale bar: 200 μm. (**E**) Histology scores of mice on day 24 of the conjunctivitis model. Data are from 3 pooled experiments (*n* = 10–20). ****P < 0.0001 by 1-way ANOVA. (**F**) Total IgE in serum. (**G**) OVA-specific IgE in serum. (**H**) Intracellular cytokine staining of cervical lymph node cells isolated from mice. Plots are gated on CD4^+^CD44^hi^ cells, and representatives of 5 mice were analyzed. Data depicted in **F**–**H** are from 1 experiment (*n* = 5) and are representative of 2 independent replicates. **P < 0.01, ***P < 0.001, ****P < 0.0001 by 1-way ANOVA.

A large-scale epidemiological study has shown that adults with AD are likely to develop selected ocular diseases in a severity-dependent manner (4). To investigate the contribution of AD to the development of conjunctivitis, we developed a potentially novel mouse model of skin inflammation–induced conjunctivitis in which mice were skin sensitized to a model antigen, OVA, on a developing AD-like skin lesion induced by daily topical treatment of MC903 in the ear to mimic AD-like skin inflammation (Figure 1A). Consistent with previous reports (18, 19), WT BALB/c mice treated epicutaneously with MC903 showed increased ear thickness compared with vehicle-treated control mice (Figure 1B). Following a 12-day rest period, the mice were challenged (vehicle or OVA) in the eye once per day for 7 consecutive days. Ophthalmologic clinical symptoms were subsequently examined and scored daily 20 minutes following the challenge. A robust and statistically significant induction in clinical scores in mice challenged ocularly with OVA (MC903 + OVA/OVA) was observed, as compared with vehicle control (MC903 + OVA/PBS; 7.7 ± 0.5 versus 2.0 ± 0.2; Figure 1C). Thus, these data indicate that skin inflammation and sensitization followed by ocular challenge induced conjunctivitis. Histopathological analysis also demonstrated that there was an accumulation of monocytes and eosinophils in conjunctival and eyelid tissues of mice challenged with OVA compared with PBS (MC903 + OVA/OVA; Figure 1, D and E). Next, we observed significantly higher expression of total IgE and OVA-specific IgE in serum of mice that developed conjunctivitis compared with controls (Figure 1, F and G). Finally, cervical lymph node cells from MC903 + OVA/OVA mice, when restimulated ex vivo with OVA, produced increased amounts of IL-4 and IL-13 but not IL-17 (Figure 1H). Taken together, these data indicate that inflammation in the skin can prime otherwise harmless antigen exposure into allergen sensitization and lead to subsequent antigen-induced conjunctivitis when challenged at a distal ocular site.

### Basophils exhibit a distinct phenotype and mediate skin inflammation–evoked conjunctivitis development.

**Figure 2:**
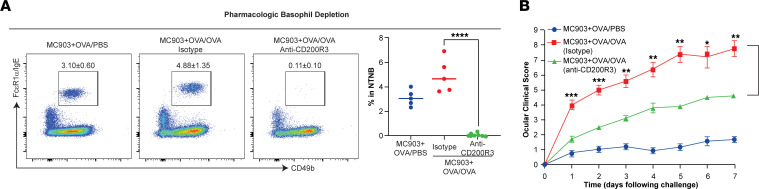
Basophils are required for conjunctivitis. (**A**) Representative flow cytometry plots and frequency of NTNB (CD3e^−^CD19^−^) CD49b^+^FcεRIα/IgE^+^ blood basophils in mice on day 24 of the conjunctivitis model. ****P < 0.0001 by 1-way ANOVA with Tukey’s post hoc test. (**B**) Clinical scores. Data depicted are from 1 experiment (*n* = 5–10) and are representative of 2 independent replicates. Results are shown as mean ± SEM, and a 2-way ANOVA with Tukey’s post hoc test was used to determine significance. *P <0.05, **P <0.01, ***P <0.001. NTNB, non-T non-B.

Dupilumab-treated patients with AD with an adverse event of conjunctivitis were found to have elevated levels of circulating basophils compared with those without the condition (17), suggesting their importance in driving ocular inflammation. Interestingly, the circulating basophil ratio in this study reached its peak around 2 or 3 months after dupilumab initiation, which coincides with the timeline of the peak of conjunctivitis (17), suggesting a role for basophils in driving conjunctivitis. Thus, we sought to investigate the role of basophils in mediating conjunctivitis in our murine skin inflammation–induced conjunctivitis model. Skin-sensitized WT BALB/c mice were treated with a mAb specific for CD200R3 to deplete basophils (20) (Figure 2A). Mice in which basophils were depleted after sensitization showed decreased OVA-evoked ocular clinical scores compared with control mAb-treated mice (4.6 ± 0.2 versus 7.8 ± 0.6, *P* < 0.01; Figure 2B). Collectively, these results indicate that basophils are key contributors to the pathogenesis of experimental conjunctivitis in mice.

**Figure 3:**
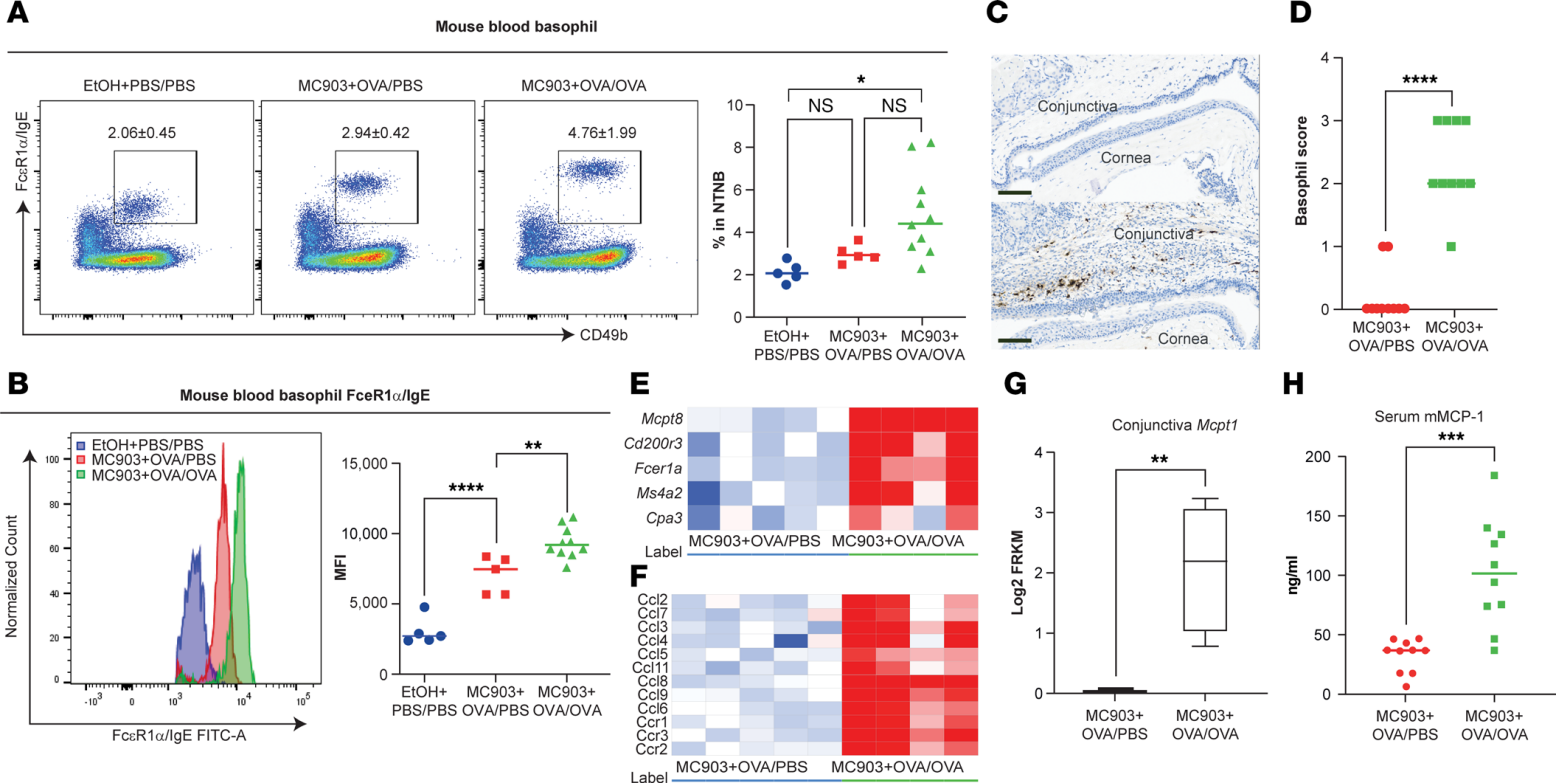
Basophils exhibit a distinct phenotype in AD-associated conjunctivitis. (**A**) Representative flow cytometry plots and frequency of NTNB (CD3e^−^CD19^−^) CD49b^+^FcεRIα/IgE^+^ blood basophils in mice on day 24 of the conjunctivitis model. Data depicted are from 1 experiment (*n* = 5–10) and are representative of 2 independent replicates. *P < 0.05 by 1-way ANOVA with Tukey’s post hoc test. (**B**) FcεRIα/IgE expression measured by MFI on blood basophils in mice on day 24 of the conjunctivitis model. Data depicted are from 1 experiment (*n* = 5–10) and are representative of 2 independent replicates. (**C**) IHC staining performed on conjunctival tissue sections at day 24 with Ab against mMCP-8 (specific for basophils). Upper, MC903 + OVA/PBS; lower, MC903 + OVA/OVA. Scale bar: 100 μm. (**D**) Basophil IHC scores. Data are from 2 pooled experiments (*n* = 10). (**E**) Heatmap of basophil signature genes. (**F**) Heatmap of chemokine and receptor signature genes. The color gradient in **E** and **F** represents fold-change values; OVA-challenged samples were compared with PBS-challenged samples (*n* = 4–5). (**G**) *Mcpt1* transcripts in conjunctival tissues (*n* = 4–5). (**H**) mMCP-1 in serum. Data depicted in **B**, **D**, **G**, and **H** are from 1 experiment (*n* = 10) and are representative of 2 independent replicates. **P < 0.01, ***P < 0.001, ****P < 0.0001 by unpaired Student’s *t* test. NTNB, non-T non-B.

To further characterize the role and phenotype of basophils in our murine experimental conjunctivitis model, we performed flow cytometry on the blood of mice. Mice sensitized with MC903 + OVA (MC903 + OVA/PBS) displayed a trend toward increased frequency of circulating basophils (Figure 3A), and these basophils expressed elevated levels of the basophil markers FcεRIα and IgE (Figure 3B) when compared with vehicle-sensitized mice (ethyl alcohol [EtOH] + PBS/PBS). Strikingly, ocular OVA challenges (MC903 + OVA/OVA) led to a trend of further increased basophil frequency (Figure 3A) and upregulation of FcεRIα and IgE compared with control mice (Figure 3B), suggesting that conjunctivitis-associated blood basophils are more responsive to IgE-mediated stimulation. In addition, OVA-challenged mice exhibited significantly increased basophil infiltration in the conjunctival tissue, as measured using IHC. This local basophil infiltrate was rarely seen in PBS-challenged mice, indicating that it was an immune response upon antigen re-exposure in conjunctival tissues (Figure 3, C and D).

Next, we identified a transcriptional signature of basophil-associated genes whose expression was greater in OVA-challenged conjunctival tissues than in PBS-challenged tissues. These genes include *Mcpt8* and *Cpa3*, 2 protease-encoding transcripts; *Fcer1*α and *Ms4a2*, which encode the α and β chains of the high-affinity IgE receptor; and *Cd200r3,* which encodes an activating receptor (21, 22) (Figure 3E). Additionally, the basophil signature transcripts from several genes encoding chemokines (*Ccl3*, *Ccl4*, and *Ccl9*) were highly expressed in OVA-challenged mice (Figure 3F), implying a role for basophils in recruiting other leukocytes to sites of inflammation. Notably, OVA challenges also induced the expression of genes involved in chemotaxis, such as basophil-directed chemokine *Ccl2* and its receptor *Ccr2* (Figure 3F), suggesting that basophils migrate out of circulation via chemotactic activity (23–25) dependent on CCL2 (possibly derived from mast cells and eosinophils).

Allergic conjunctivitis in humans is defined as a disease associated with IgE-mediated mast cell activation in conjunctival tissue, leading to the release of preformed mediators including histamine and proteases (26). We observed an increase in the mast cell protease-1 (*Mcpt1*) conjunctival transcript level (Figure 3G) and serum mouse mast cell protease-1 (mMCP-1) protein expression (Figure 3H) in OVA-challenged mice, suggesting mast cell degranulation may also contribute to conjunctivitis in this model.

### CD4^+^ T cells mediate antigen-specific skin inflammation–evoked conjunctivitis development.

**Figure 4:**
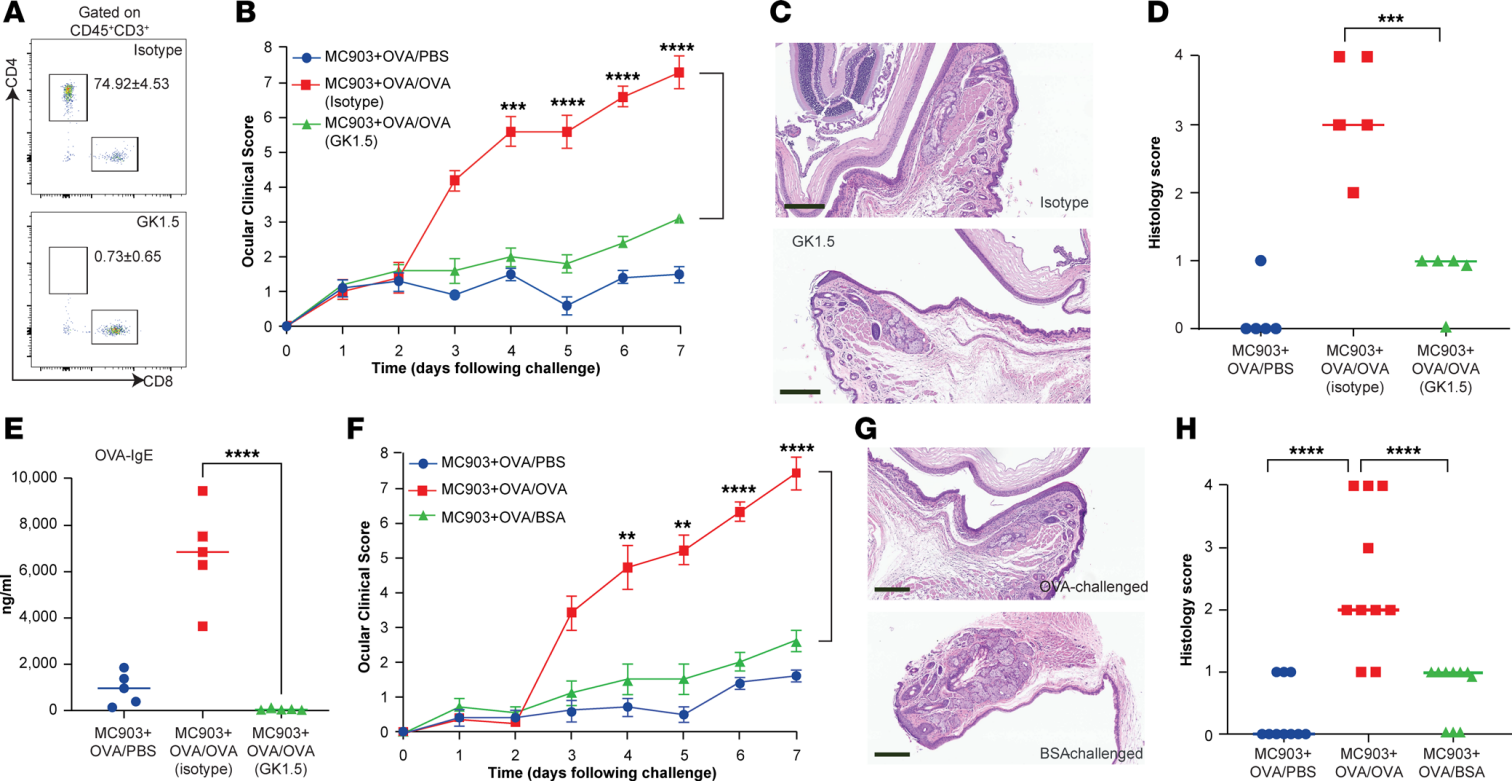
CD4^+^ T cell requirement and antigen specificity in skin inflammation–induced conjunctivitis. (**A**) Representative flow cytometry plots showing CD4^+^ T cell depletion in the blood. (**B**) Clinical signs. Data depicted are from 1 experiment (*n* = 5) and are representative of 2 independent replicates. Results are shown as mean ± SEM, and a 2-way ANOVA with Tukey’s post hoc test was used to determine significance. (**C**) Representative H&E histopathology images. Upper, isotype; lower, GK1.5. Scale bar: 200 μm. (**D**) Histology scores of mice on day 24 of the conjunctivitis model. Data depicted are from 1 experiment (*n* = 5) and are representative of 2 independent replicates. (**E**) OVA-specific IgE in serum. Data depicted are from 1 experiment (*n* = 5) and are representative of 2 independent replicates. (**F**) Clinical signs. Data depicted are from 1 experiment (*n* = 5) and are representative of 2 independent replicates. Results are shown as mean ± SEM, and a 2-way ANOVA with Tukey’s post hoc test was used to determine significance. (**G**) Representative H&E histopathology images. Upper, MC903 + OVA/OVA; lower, MC903 + OVA/BSA. Scale bar: 200 μm. (**H**) Histology scores of mice on day 24 of the conjunctivitis model. Data are from 2 pooled experiments (*n* = 10). Data depicted are from 1 experiment (*n* = 5) and are representative of 2 independent replicates. **P < 0.01, ***P < 0.001, ****P < 0.0001.

Central to the pathogenesis of allergic disorders and conjunctivitis are CD4^+^ T cells. Clonally expanded pathogenic effector T_H_2 cells, defined by sharing a common α- or β-chain complementarity-determining region 3, are detected in both inflamed tissues and peripheral blood from patients with type 2 inflammation, consistent with an antigen-specific response (27). The role of CD4^+^ T cells in experimental conjunctivitis was assessed in mice treated with a depleting anti-CD4 Ab (GK1.5) 6 days prior to ocular OVA challenge and again on the day of challenge. Treatment with GK1.5 led to a complete loss of CD4^+^ T cells (Figure 4A) as well as a dramatic reduction in ocular clinical (3.1 ± 0.3 versus 7.3 ± 1.5, *P* < 0.0001) and histology scores and in OVA-specific IgE in serum (Figure 4, B–E). These data demonstrate that CD4^+^ T cells are required for the induction of ocular inflammation following skin sensitization.

To test the antigen specificity of our experimental conjunctivitis model, mice were sensitized in the skin with MC903 + OVA, and then challenged in the eye with a second antigen, BSA. Indeed, mice that were OVA-sensitized and then exposed to ocular BSA displayed a lack of ocular inflammation (Figure 4, F–H). These data reveal that the conjunctivitis observed in this mouse model is antigen specific.

### IL-4Rα blockade partially alleviates conjunctivitis development.

**Figure 5:**
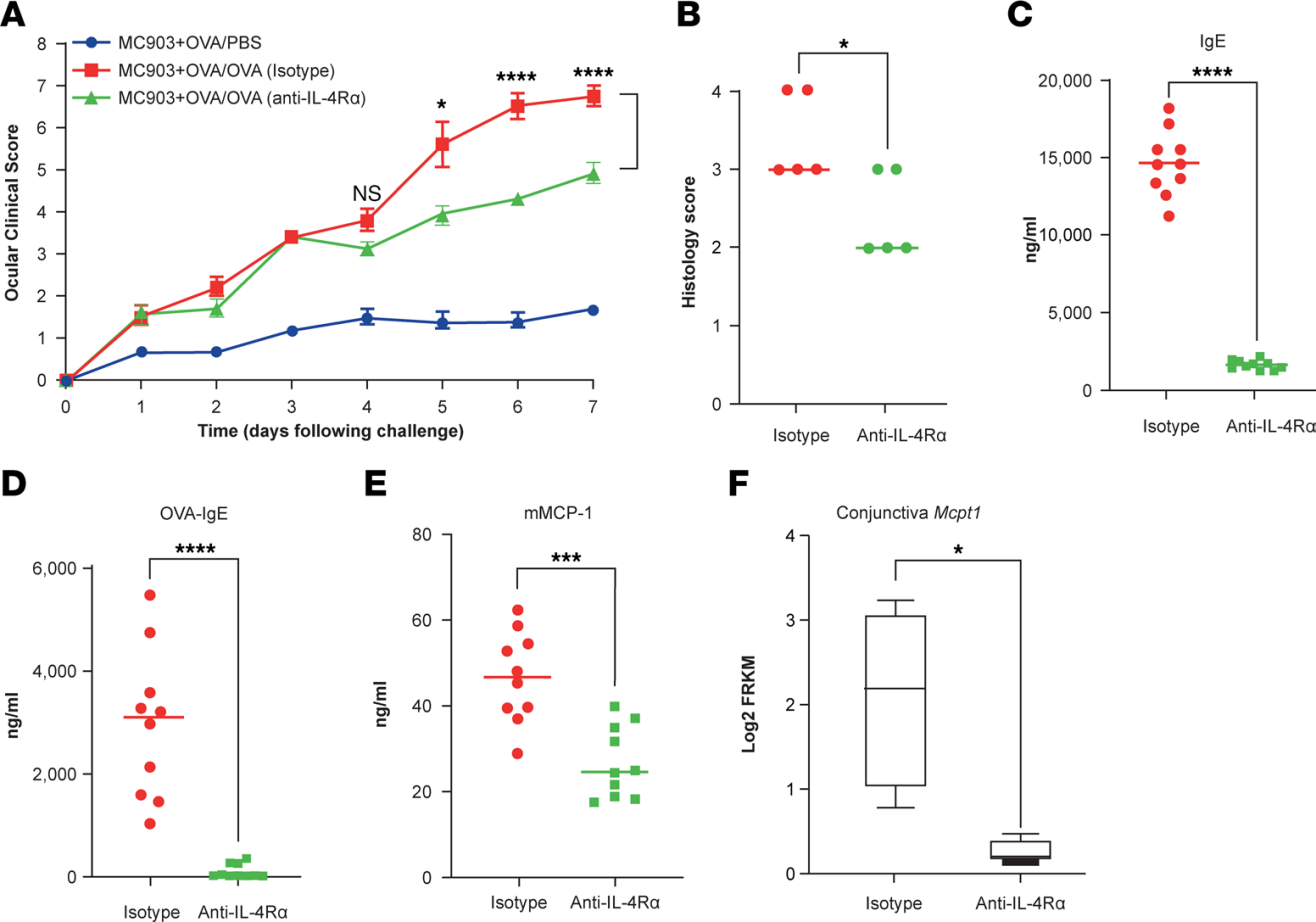
IL-4Rα blockade partially alleviates conjunctivitis development. (**A**) Clinical signs. Data depicted are from 1 experiment (*n* = 5) and are representative of 2 independent replicates. Results are shown as mean ± SEM, and a 2-way ANOVA with Tukey’s post hoc test was used to determine significance. (**B**) Histology scores. Data depicted are from 1 experiment (*n* = 5) and are representative of 2 independent replicates. (**C**) Total IgE in serum. (**D**) OVA-specific IgE in serum. (**E**) mMCP-1 in serum. Data depicted in **C**–**E** are from 1 experiment (*n* = 10) and are representative of 2 independent replicates. (**F**) *Mmcp1* transcripts in conjunctival tissues. *n* = 4. *P < 0.01, ***P < 0.001, ****P < 0.0001 by unpaired Student’s *t* test for **B–F**.

Next, we aimed to dissect the possible mechanisms of increased frequency of conjunctivitis in patients with AD treated with dupilumab by exploring the impact of IL-4Rα signaling in our model (4, 7, 13). The dupilumab surrogate Ab was used to block IL-4Rα following induction of skin inflammation with MC903 + OVA, and anti–IL-4Rα–treated mice ultimately displayed partially attenuated ocular inflammation compared with isotype-treated mice (4.9 ± 0.7 versus 6.7 ± 0.7, *P* < 0.0001; Figure 5A). In addition, the serum levels of total IgE, OVA-specific IgE, and mMCP-1 were markedly attenuated after anti–IL-4Rα treatment (Figure 5, B–E). Similarly, *Mmcp1* transcript levels in conjunctival tissues were decreased following anti–IL-4Rα treatment (Figure 5F). We concluded the functional role of IL-4Rα signaling in the development of conjunctivitis in response to antigen sensitization and challenge of mice. 

### IL-4Rα blockade attenuates basophil activation.

**Figure 6:**
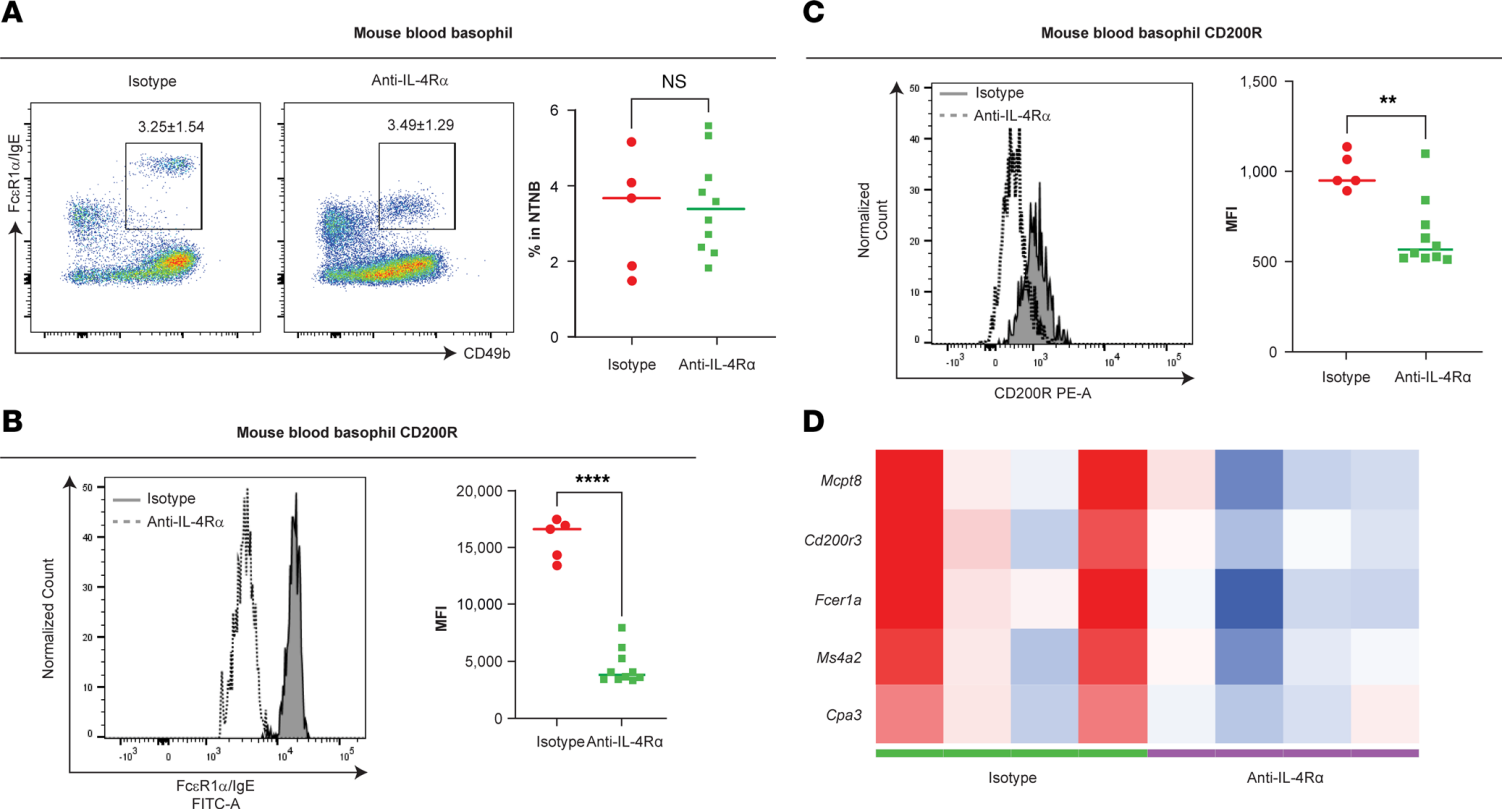
IL-4Rα blockade attenuates basophil activation. (**A**) Representative flow cytometry plots and frequency of NTNB (CD3e^−^CD19^−^) CD49b^+^FcεRIα/IgE^+^ blood basophils in mice on day 24 of the conjunctivitis model. (**B**) FcεRIα/IgE expression measured by MFI on blood basophils in mice on day 24 of the conjunctivitis model. (**C**) CD200R expression measured by MFI on blood basophils in mice on day 24 of the conjunctivitis model. Data depicted are from 1 experiment (*n* = 5–10) and are representative of 2 independent replicates. **P < 0.01, ****P < 0.0001 by unpaired Student’s *t* test for **B** and **C**. (**D**) Heatmap of basophil signature genes. The color gradient represents fold-change values; OVA-challenged samples were compared with PBS-challenged samples. *n* = 4. NTNB, non-T non-B.

Since basophils were required for the development of skin inflammation–induced conjunctivitis in mice (Figure 2), we then tested whether targeting IL-4Rα signaling after skin sensitization could affect basophils. Mice receiving anti–IL-4Rα treatment did not display differences in the frequency of circulating basophils (Figure 6A). However, we observed downregulation of the murine basophil activation marker, CD200R, and FcεRI/IgE compared with mice receiving isotype control (Figure 6, B and C). Following IL-4Rα Ab blockade, we also observed decreased expression of basophil marker genes (e.g., *Mcpt8*, *Cpa3*, *Cd200r3*, *Fcer1a*, and *Ms4a2*) (Figure 6D). These findings prompt the hypothesis that IL-4Rα signaling pathway promotes basophil responding to IgE stimulation and activation (28); and IL-4Rα blockade likely decreased basophils’ activation by hindering IgE production. Our results showed that IL-4Rα signaling contributes to conjunctivitis development in the context of skin inflammation by producing antigen-specific IgE Abs and activating mucosal mast cells (Figure 5, D and E) and basophils (Figure 6).

### Anti–IL-4α–attenuated and –augmented gene profiles in the conjunctivitis model.

**Figure 7:**
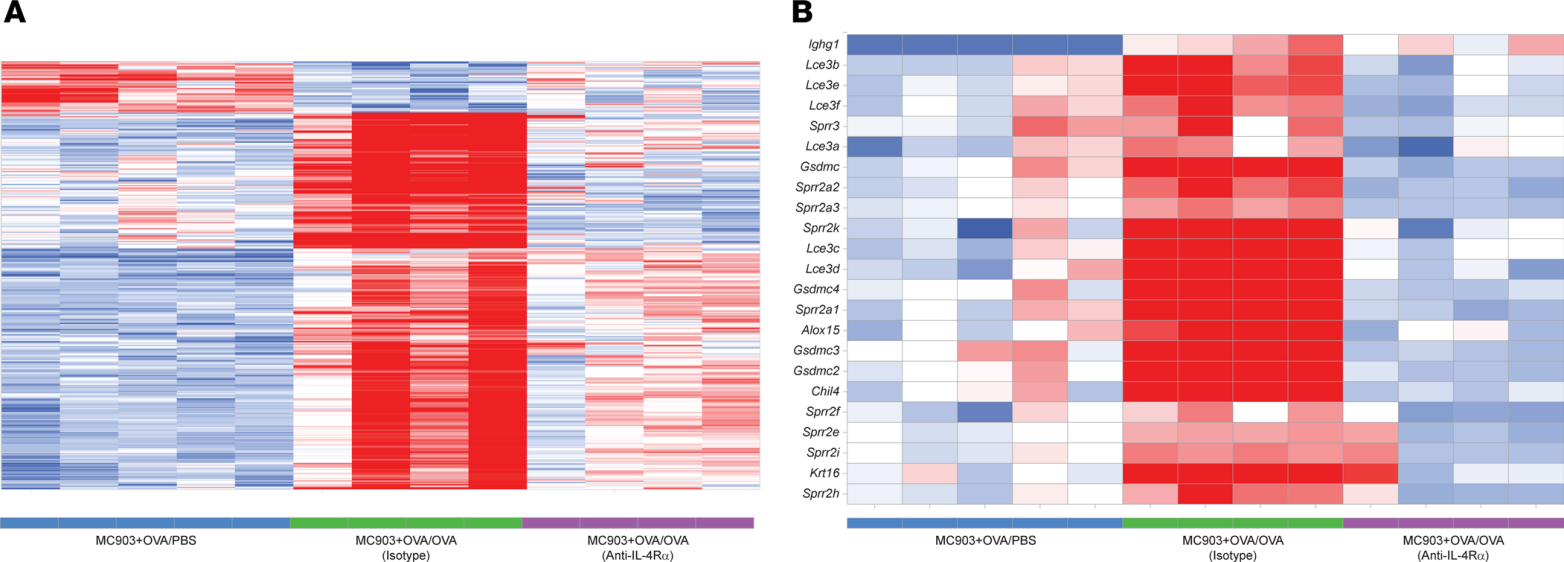
IL-4Rα blockade resulted in a preferential, strong normalization of the gene expression pattern. (**A**) Hierarchical clustering of disease signature genes. 352 disease signature genes were defined by using the General Linear Model function in Array Studio and comparing groups MC903 + OVA/OVA (isotype control) versus MC903 + OVA/PBS then applying a fold-change cutoff of ± 1.5 and an FDR less than 0.05. (**B**) Heatmap of selected anti-IL4-Rα–attenuated genes. Select genes were highlighted based on prior knowledge of their importance as biomarkers in type 2 inflammation. Color gradient represents the *Z* score of normalized gene expression across samples. *n* = 4–5.

**Figure 8:**
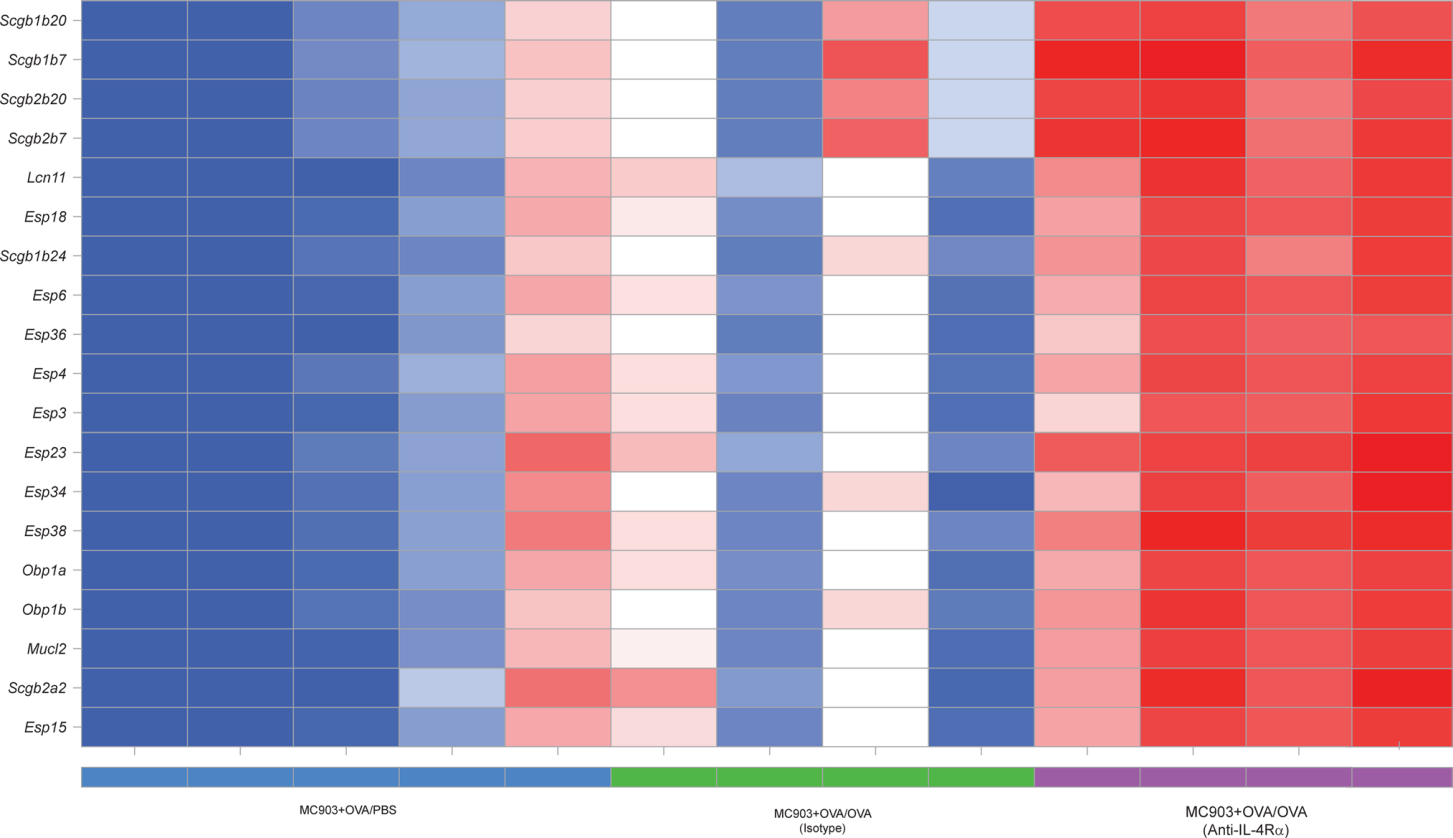
IL-4Rα–augmented conjunctival tissue gene expression analysis. Heatmap of select top upregulated genes found to be increased upon anti–IL-4Rα treatment were identified by comparing groups MC903 + OVA/OVA (anti–IL-4Rα) versus MC903 + OVA/OVA (isotype control) using the General Linear Model in Omicsoft and applying an FDR cutoff of less than 0.05. Select genes were highlighted based on prior knowledge of their importance as biomarkers in ocular disorders. Color gradient represents the *Z* score of normalized gene expression across samples. *n* = 4–5.

To characterize conjunctivitis and the effects of IL-4Rα signaling at the transcriptomic level, we performed RNA-Seq on conjunctival tissues in OVA-challenged mice that received isotype or anti–IL-4Rα treatment, as well as in control mice that were ocularly challenged with PBS (MC903 + OVA/PBS) (Figure 7). Analysis of gene expression in the conjunctival tissues of OVA-challenged versus PBS-challenged mice showed differentially regulated genes or pathways. *Krt16*, a marker of keratinocyte activation (29), was elevated, suggesting that epithelial damage is an important factor in causing the pathophysiology of conjunctivitis. Late differentiation genes (*Sprr2* and *Lce3*) (30, 31) and macrophage-derived chitinase *Chil4* (32) were also elevated in the conjunctivitis model (Figure 7B). We then discovered preferential, strong normalization of ocular gene expression in mice treated with anti–IL-4Rα (Figure 7, A and B). Thus, blockade of IL-4Rα resulted in the normalization of a unique ocular gene expression pattern compared with isotype control. In addition, we identified IL-4Rα–augmented pathways by comparing blockade of IL-4Rα with isotype control, which may underlie the partial efficacy in this model and could possibly represent candidate mediators of the paradoxically increased frequency of conjunctivitis following dupilumab in patients with AD. Furthermore, a statistically significant increase was shown for genes that encode secretoglobin (*Scgb*), mammoglobin (*Scgb2a2*), lipocalin (*Lcn11*), exocrine gland-secreted peptides (*Esp*), and mucin-like protein 2 (*Mucl2*) after anti–IL-4Rα treatment (Figure 8). These findings are consistent with peptide profiles in human tears from patients affected by vernal keratoconjunctivitis (33). A lacrimal gland autoantigen that is associated with lacrimal gland autoimmunity and ocular surface sequelae (34), odorant binding protein 1a (*Obp1a*), was also elevated by anti–IL-4Rα treatment (Figure 8).

## Discussion

In the present study, we describe and characterize a potentially novel mouse model in which skin sensitization to a model antigen followed by ocular antigen challenge results in experimental conjunctivitis. Epidemiologic data have demonstrated that adults with AD have a significant and disease-severity–dependent increased risk of developing select ocular diseases, and, thus, our preclinical data expand upon the concept of “atopic march,” which describes this developmental progression of atopic diseases (1, 3). The mouse model presented herein serves to support this theory and is the first to describe atopic march leading to conjunctivitis in a preclinical setting. Our model demonstrates that sensitization to antigen, in the presence of MC903 (an agent that induces AD-like inflammation) in the skin leads to antigen-specific conjunctivitis when challenged at ocular sites. This model broadens our mechanistic understanding of atopic march by identifying additional immunological factors.

We demonstrated that basophils and CD4^+^ T cells were required for the development of conjunctivitis in mice, as depleting them following the sensitization phase limited the disease. Recent studies suggest basophils are the key effector cell to induce type 2 inflammation and itch in response to allergens (19, 35). Basophils are predominantly found in the blood and generally not present in tissues in the steady state (36). MC903-evoked skin inflammation has been shown to be associated with exaggerated thymic stromal lymphopoietin (TSLP) production during sensitization (18), which makes basophils more responsive to IgE-mediated stimulation, suggesting this mechanism may play a role in our current studies (35). Upon exposure to OVA via eye drops, basophils were recruited into inflamed conjunctival tissues and further upregulated activation markers with an enhanced capacity to mediate an antigen-induced reaction in the setting of AD-associated conjunctivitis (Figure 3) (37). Basophils may play a pathophysiological role in the development of murine conjunctivitis through the release of inflammatory mediators such as histamine and leukotriene C4 (LTC4) in response to IgE-mediated activation, contributing to immediate hypersensitivity reactions (36). In addition, sensory neurons, which are strongly associated with inflammatory itch, might also be activated by basophil mediators (35). Taken together, these findings suggest that conjunctivitis-associated inflammation enhances the activation of basophils (and likely mast cells) to mediate antigen-induced response. While our current study focused on basophils and T cells, we hypothesize that they are likely not the only cellular mediators. Indeed, an increase in eosinophils and monocytes were present in conjunctival tissues as assessed histopathologically following ocular antigen challenge. Thus, future studies will be required to further investigate other cellular mechanisms that promote conjunctivitis.

Depletion of CD4+ T cells in our model revealed a complete dependency of conjunctivitis development following skin inflammation on this cell type, as evidenced by complete amelioration of ocular clinical scores. It is still unclear how the local cutaneous sensitization and T_H_2 hyperreactivity lead to systemic effects and eventually promote an allergic response in the conjunctival tissues. Patients with AD have elevated pathogenic effector T_H_2 cells in their peripheral blood with increased IL-13 expression, indicating a systemic T_H_2 environment (38). A significant fraction of peripheral pathogenic effector T_H_2 clonotypes is reactive to disease-associated antigens and subsequently migrates to the conjunctival tissues upon topical challenge with said antigen. However, the homing mechanism is unknown. The interaction between the T_H_2 cell-expressed chemoattractant receptor and its ligand by the conjunctival epithelium needs to be identified to better understand specific T cell homing to the inflamed ocular tissues (27).

Higher rates of unspecified and allergic conjunctivitis in patients with AD who received dupilumab, tralokinumab, or lebrikizumab treatment have prompted efforts in the medical community to identify potential underlying pathogenic mechanisms (10, 11, 39). Currently, our knowledge base lacks clarity regarding the pathogenesis of conjunctivitis and why its appearance is nearly exclusive to patients with AD. To complement ongoing phase IV clinical studies to investigate the phenotype and ocular biomarkers of conjunctivitis in patients (40), we used what we believe to be a novel skin inflammation–evoked conjunctivitis model to further investigate potential effects of IL-4Rα inhibition in mice. The partial efficacy of the dupilumab surrogate Ab was most likely not due to incomplete inhibition of the pathway, since we observed a near elimination of IgE, indicative of full pharmacological target inhibition. We speculate the existence of additional mechanisms besides IL-4Rα signaling that mediate conjunctivitis, although a possible mechanism occurring at the epithelial layer of the eye may also become exacerbated by anti–IL-4Rα treatment. We then used RNA-Seq to define a disease signature and to assess the effect of blocking IL-4Rα at the transcriptomic level, resulting in the discovery of a disease signature that can be attenuated by anti–IL-4Rα. Pathways that are increased in disease and attenuated with anti–IL-4Rα involve T_H_2 signaling as well as epithelial damage. Genes implicated in epidermal diseases and dry eye, such as *Krt16* and *Sprr2* (30, 31), are downregulated with anti–IL-4Rα treatment. Thus, dupilumab surrogate Ab treatment resulted in modulation of IL-4Rα–regulated genes in the conjunctival tissue, likely contributing to the partial attenuation of allergic conjunctivitis.

Our present observations on the therapeutic effect of IL-4Rα blockade in murine experimental conjunctivitis are inconsistent with the increased frequency of conjunctivitis following treatment with dupilumab in patients with AD. Indeed, in accordance with a suggested therapeutic role for IL-4Rα/basophil signaling in our present studies, recent clinical data from a single small study have suggested an increase in basophils associated with conjunctivitis in patients with AD following dupilumab treatment (17). However, conjunctivitis comprises a heterogeneous group of clinicopathologic conditions with a wide variety of signs and symptoms (41). Diagnosis of conjunctivitis following dupilumab treatment in clinical trials was typically performed by dermatologists and allergists without an ophthalmologist’s evaluation. As a result, conjunctivitis cases reported include a variety of subtypes without distinguishing between their classifications (9). A subtype of patients with AD may have a predominantly allergic phenotype of conjunctivitis and, thus, the therapeutic effects of IL-4Rα blockade we observed in our preclinical model could reflect this disease phenotype. Indeed, our data provide support for potential efficacy of dupilumab in an ongoing Phase 2 study to evaluate the efficacy of dupilumab in the treatment of atopic keratoconjunctivitis (42). Finally, we cannot reflect the full range of symptoms reported in humans in preclinical models, so the therapeutic effects of anti–IL-4Rα that we observed in a preventive setting might not translate to long-term intervention in certain established chronic diseases. Potential discrepancies between mouse and human biology must be considered.

Despite these limitations, our research has shed light upon the potential pathomechanism of conjunctivitis in AD trials by identifying anti–IL-4Rα-augmented genes that could account for partial efficacy of IL4Ra blockade on murine conjunctivitis, as well as candidate markers for conjunctivitis clinically. These genes encode for a wide range of biological categories, such as mammaglobin, lipocalin, and secretoglobin, suggesting a possible mechanism occurring at the epithelial layer of the eye that may become exacerbated by anti–IL-4Rα treatment and could underlie conjunctivitis in the clinic. In human studies, levels of these proteins were found to be significantly overexpressed in vernal keratoconjunctivitis tear samples compared with the control group, correlating to the severity of the disease. In particular, when patients were treated with topical cyclosporine or corticosteroids, their tear samples indicated significantly lower protein levels (33). Lacrimal drainage impairment has also recently been associated with conjunctivitis in a small number of patients with AD following dupilumab treatment, suggesting that lacrimal gland-derived enzymes and tear proteins may play a role in chemical communication and immunity (43).

In summary, we have described and characterized a potentially novel murine model of skin inflammation–evoked conjunctivitis. Our research also identified a potential pathogenic mechanism involved in conjunctivitis in AD trials and shed light upon this new entity of conjunctivitis. Further studies characterizing the biological role of anti–IL-4Rα–augmented pathways that we identified in the mouse model, especially in patients with AD before and during dupilumab treatment, would further reveal our understanding of conjunctivitis.

## Methods

### Mice.

Female BALB/c mice were purchased from the Jackson Laboratories. All mice were used between 9 and 12 weeks of age, and all experiments employed age- and gender-matched controls to account for any variations in data sets compared across experiments. Mice were bred and housed in specific pathogen-free conditions at Sanofi.

### Reagents and treatments.

Mice were treated daily with 2.5 nmol MC903 (calcipotriol; Tocris Bioscience) in 25 μL of 100% EtOH applied to the right ear for 7 days. As a vehicle control, the same volume of EtOH was applied. On days 1, 3, and 5, 10 μg of OVA (A7642; Sigma-Aldrich) was injected intradermally in a 20 μL volume of sterile PBS by inserting the needle into the superficial dermis in the ear as close to the epithelium as possible. On day 6, 10 μg of OVA was applied to the same ear in 5 μL of PBS. Challenge via topical OVA or BSA (Sigma-Aldrich) instillation (250 μg/10 μL eye drop) had been administered once daily for 7 days. Clinical scoring was performed 20 minutes after challenge and was done once daily from day 18 to day 24. Mice were examined on the basis of 4 independent parameters, which include eyelid edema, secretion, squinting, and redness. Each parameter was ascribed 0 (i.e., absent) to 3+ points (i.e., maximal) and was summed to yield a maximum score of 12+. For CD4^+^ T cell depletion, animals were i.p. injected on days 12 and 18 with 200 μg of anti-CD4 Ab (clone GK1.5, BioXCell) or 200 μg rIgG2b (BioXCell) in a total volume of 200 μL PBS. For pharmacologic IL-4Rα blockade, WT mice received i.p. injections of 200 μL of purified anti-mouse IL-4Rα Ab (REGN1103, a dupilumab mouse homologue; gift from Regeneron) or isotype control (REGN2390-L1; gift from Regeneron) on days 10, 14, 18, and 21 dosed at 25 mg/kg. For pharmacologic depletion of basophils, WT mice received i.p. injections of 100 μL of purified anti-mouse CD200R3 Ab (0.4 mg/mL; clone Ba160, BioLegend) or rat IgG2b, κ isotype control (BioXCell) every other day from day 15 to day 23. The effect of depletion was confirmed by assessing blood basophil levels using flow cytometry on the last day of the model.

### Skin inflammation assessment.

To assess mouse ear skin inflammation induced by MC903, ear thickness was measured daily with dial calipers.

### ELISA.

To measure mouse serum protein levels, 0.5 to 1 mL of blood was collected into 1.5 mL microcentrifuge tubes and was allowed to clot for 60 minutes at room temperature. Tubes were then centrifuged for 10 minutes at 1,000 *g* at 4°C. Sera were collected and stored at –80°C until proteins were quantified according to the manufacturer’s instructions using enzyme-linked immunoassay kits for the mouse OVA IgE (Cayman), total IgE (Invitrogen), and mMCP-1 (Invitrogen).

### Cell culture.

Cervical lymph node cells were isolated and cultured in RPMI medium with 10% FCS, penicillin, and streptomycin and with 100 μg/mL OVA for 72 hours. Cells were stimulated with phorbol myristate acetate and ionomycin in the presence of brefeldin A for 4–5 hours. The cells were harvested and analyzed for cytokine production, and further analysis was performed using FlowJo software (Tree Star).

### Flow cytometry.

For animal studies, 50–100 μL of blood was collected into ethylenediaminetetraacetic acid-coated tubes, followed by red blood cell lysis using red blood cell lysis buffer (Sigma-Aldrich) at room temperature for 25 minutes and washed with PBS once. All cells were stained with viability dye (1:1,000; BD Horizon) for viability on ice for 20 minutes, followed by primary Abs on ice for 30 minutes prior to data acquisition on a BD Fortessa (BD Biosciences). Basophils were defined as live CD49b^+^FcεRIα/IgE^+^ cells that were negative for expression of CD3e and CD19. The mouse basophil canonical activation marker CD200R was also stained. All flow cytometry data were analyzed with FlowJo v10 software (Tree Star).

### Histology.

Mice were euthanized 1 hour after the final challenge. Once removed, whole heads were fixed in neutral-buffered formalin (NBF) for at least 24 hours. Conjunctiva and surrounding skin were then removed and fixed for a minimum of 48 additional hours prior to paraffin processing and embedding. Tissue sections 5 μm thick were collected and stained with H&E (44). Histopathology of the conjunctiva was scored according to the amount of inflammatory infiltrate present. Conjunctiva that did not show any infiltrate was scored as a 0. If rare inflammatory cells were present, the tissue was assigned a score of 1. When infiltrate was more noticeable but still mild, the conjunctiva was scored as 2. Conjunctiva was scored as a 3 if infiltrate was present in multifocal to coalescing clusters. Finally, if infiltrate was severe and diffusely present throughout the conjunctiva, a score of 4 was assigned.

### IHC staining.

IHC was performed using anti–mMCP-8 (clone TUG8, BioLegend). Isotype control was performed using rat IgG2a (Bio-Rad Laboratories). The scoring of basophils in conjunctival tissues followed a similar pattern to the histology evaluation above: conjunctiva that did not show any basophils was scored as a 0; if trace amounts of basophils were present, the tissue was assigned a score of 1; when more noticeable basophils were present, the conjunctiva was scored as a 2; finally, conjunctiva tissues were scored as a 3 if basophils manifested in multifocal to coalescing clusters.

### RNA isolation and transcriptome sequencing.

Conjunctival tissue samples were collected into tubes containing RNAlater and were later homogenized using QIAzol lysis reagent (Qiagen) and ceramic beads (Precellys). Phase-lock tubes (Quantabio) were used to assist separation following chloroform-based phase separation of RNA before total RNA was extracted with the RNeasy Lipid Tissue Mini Kit (Qiagen). RNA quantification and quality assessment were performed with Nanodrop 1,000 and RNA 6,000 Nano (Agilent Bioanalyzer). Total RNA was diluted to 1 μg in 25 μL of RNase-free water prior to a polyA selection using Oligo(dT) beads from Illumina’s Stranded mRNA Prep Ligation kit. Enriched mRNA species were converted to full length complementary deoxyribonucleic acid, dual indexed, and then amplified using 10 PCR cycles. The final libraries were assessed using the D1000 kit on the TapeStation (Agilent) for quality as well as the High Sensitivity DNA Qubit for quantification. To prepare for sequencing, libraries were pooled together at equal molar before denaturing with sodium hydroxide and diluted to 1.5 pM in accordance with Illumina’s denaturation protocol for the NovaSeq6000. Sequence runs were performed on a NovaSeq6000 V1.5 SP flow cell and a 2′ 90 bp paired-end run.

### RNA-Seq data analysis.

Data analysis was completed using Omicsoft’s Array Studio. On average, each sample was sequenced at a depth of approximately 75 million paired-end reads. Illumina adaptors were trimmed during the binary base call (BCL) to FastQ conversion. All raw data was checked for quality using the “Raw Data QC Wizard” function within Array Studio. Poor quality reads were filtered out using a Q score cutoff of 20. Following the above filter criteria, about 58 million paired-end reads per sample were uniquely mapped to the B38 Mouse Reference Genome using the Ensembl.R100 Gene Model. Raw counts were then converted to fragments per kilobase of transcript per million mapped reads (FPKM) using the “Report Gene/Transcript Counts” function in Array Studio. Low counts were filtered using a cutoff of 10 in at least 5 samples (*n* = 5 for each group) then normalized using the 75th quantile. A constant of 1 was added to all normalized FPKM data prior to a Log_2_ transformation. This normalized and transformed data was used in all downstream analysis, including principal component analysis (PCA), hierarchical clustering, heatmaps, *t* tests, and Ingenuity Pathway Analysis. Outliers (a total of 2) were confirmed using model-based outlier detection, correlation-based quality check, and PCA plots, as well as hierarchical clustering (unsupervised) within Array Studio. *t* tests were generated using the General Linear Model function in Array Studio. Pathway analysis was completed using fold change and *P* values obtained via *t* test and imported into Qiagen Ingenuity. RNA-Seq data that support the finding of this study have been deposited with the Gene Expression Omnibus (GEO) repository under accession number GSE217899.

### Statistics.

All statistical analyses were performed using GraphPad Prism 9. Unless otherwise indicated, all statistical tests are 1-way ANOVA with Tukey’s post hoc test. *P* ≤ 0.05 was considered significant.

### Study approval.

All experiments were performed under the Sanofi IACUC–approved protocols and in accordance with its guidelines.

## Author contributions

HH developed the study, designed and performed the experiments, and wrote the manuscript. SC, JJ, KTCS, GQ, JG, SS, JE, EZ, and RB performed the experiments. DB provided scientific oversight of histology and IHC work. KTCS, DB, PB, and AH provided intellectual input. KTCS and AH contributed to manuscript editing.

## Version 1 01/10/2023

In-Press Preview

## Version 2 02/08/2023

Electronic publication
